# Stressful conditions reveal decrease in size, modification of shape but relatively stable asymmetry in bumblebee wings

**DOI:** 10.1038/s41598-018-33429-4

**Published:** 2018-10-11

**Authors:** Maxence Gerard, Denis Michez, Vincent Debat, Lovina Fullgrabe, Ivan Meeus, Niels Piot, Ombeline Sculfort, Martin Vastrade, Guy Smagghe, Maryse Vanderplanck

**Affiliations:** 1 Laboratoire de Zoologie, Research institute of Biosciences, University of Mons, Place du Parc 23, 7000 Mons, Belgium; 2 Institut de Systématique, Evolution, Biodiversité, ISYEB, UMR 7205 CNRS MNHN UPMC EPHE, Muséum national d’Histoire naturelle, Sorbonne Universités, CP 50, 45 rue Buffon, 75005 Paris, France; 3 Laboratory of Numerical Ecology of Aquatic Systems, Research institute of Biosciences, University of Mons, Place du Parc 23, 7000 Mons, Belgium; 4 Department of Crop Protection, Faculty of Bioscience Engineering, Ghent University, Coupure Links 653, B-900 Ghent, Belgium; 5 Laboratory of Evolutionary Genetics and Ecology, Research Unit in Environmental and Evolutionary Biology, Namur Research Institute for Life Sciences, University of Namur, 5000 Namur, Belgium

## Abstract

Human activities can generate a wide variety of direct and indirect effects on animals, which can manifest as environmental and genetic stressors. Several phenotypic markers have been proposed as indicators of these stressful conditions but have displayed contrasting results, depending, among others, on the phenotypic trait measured. Knowing the worldwide decline of multiple bumblebee species, it is important to understand these stressors and link them with the drivers of decline. We assessed the impact of several stressors (i.e. natural toxin-, parasite-, thermic- and inbreeding- stress) on both wing shape and size and their variability as well as their directional and fluctuating asymmetries. The total data set includes 650 individuals of *Bombus terrestris* (Hymenoptera: Apidae). Overall wing size and shape were affected by all the tested stressors. Except for the sinigrin (e.g. glucosinolate) stress, each stress implies a decrease of wing size. Size variance was affected by several stressors, contrary to shape variance that was affected by none of them. Although wing size directional and fluctuating asymmetries were significantly affected by sinigrin, parasites and high temperatures, neither directional nor fluctuating shape asymmetry was significantly affected by any tested stressor. Parasites and high temperatures led to the strongest phenotype modifications. Overall size and shape were the most sensitive morphological traits, which contrasts with the common view that fluctuating asymmetry is the major phenotypic marker of stress.

## Introduction

A large number of habitats and wildlife are under threat, especially due to human activities and many techniques have been developed to measure the potential stressors^1^. Indeed, the detection of populations undergoing such stresses has become a central point in conservation biology since it allows applying accurate conservation plans^2^. Numerous phenotypic markers have been proposed to detect these stresses^3,4^. Among them, fluctuating asymmetry (FA) is used to quantify the developmental stability under various conditions^5^. Developmental stability refers to the developmental processes that ensure phenotypic consistency under fixed environmental and genetic conditions in spite of small random variation during development (i.e. developmental noise)^6–8^. From the postulate that both sides of a symmetrical organism are controlled by the same genes and are exposed to the same environmental conditions, it is considered that deviations from perfect symmetry reflect those random errors of the development, and the ability of the organism to buffer them^5^. Increase in FA is thus usually interpreted as a decrease in developmental stability. In some field and laboratory studies, fluctuating asymmetry has been shown to increase under a range of both genetic and environmental stresses (e.g. inbreeding, low habitat connection, toxic stress; e.g.^9,10^). However, other studies have failed to detect such relation with stress e.g.^11^. The use of FA as a general and sensitive stress indicator has thus been seriously questioned^12,13^, see^14^ for a quantitative historical survey of the literature. Directional asymmetry (DA) is another type of asymmetry, which implies a predominant direction and a population mean of the left-right asymmetry different from zero^5^. Surprisingly enough, DA is common among insect wings^15^. This type of asymmetry has been thought to be adaptive and genetically determined^16^ but see^15^. Besides asymmetry, shift in overall wing shape, size and increase in trait variances have been reported under stressful conditions and have then been proposed as stress indicators^17–19^, although they have been very poorly investigated.

By pollinating major crops (e.g. tomato, oilseed rape) and various wild plants (e.g. Fabaceae), bumblebees show high economic and ecological value^20^. It has been demonstrated that their populations are suffering from a severe decline for the last decades; with potentially serious consequences on the ecosystem services they provide^21,22^. Five main factors are traditionally pinpointed: (i) modification and fragmentation of high quality habitats, (ii) agricultural practices, (iii) pathogen parasite spillover, (iv) competition with alien species, and (v) climate change reviewed in^23^. All these factors and their potential synergy lead to significant direct or indirect environmental and genetic stresses that have potentially lethal and sub-lethal effects on bumblebee individuals.

Here, we investigate the effect of a high diversity of distinct stressors, which are known to affect pollinators, on the size and shape of bumblebee anterior wings (*Bombus terrestris*; Hymenoptera: Apidae) reared in laboratory conditions. In bee populations under stabilizing selection, phenotypic shifts can negatively affect life history traits related to fitness, for example, mating success in honeybees^24^ and foraging range^25^. We tested four different stressors: (i) nutritional stress using a willow (i.e. *Salix* sp.) diet supplemented with plant secondary compounds, either sinigrin (i.e. glucosinolate found in some Brassicaceae) or amygdalin (i.e. cyanogenic glycoside found in Fabaceae and Rosaceae) at 100% and 200% of their natural concentration; (ii) parasite stress using the parasitic neogregarine *Apicystis bombi* (Apicomplexa: Neogregarinorida)*;* (iii) thermic stress comparing rearing temperatures of 21 °C and 33 °C; and (iv) an inbreeding stress using F1 inbred generations. For each treatment, a control was established using non-supplemented *Salix* diet, a rearing temperature of 26 °C. We hypothesised that those stresses should lead to a significant shift in overall wing size and shape as well as to a higher trait variability and fluctuating asymmetry. Since fluctuating asymmetry displayed very contrasting results in previous studies, shifts in overall size and shape might turn out to be more accurate phenotypic markers of stress.

## Results

### Wing size and shape analyses

**Figure 1:**
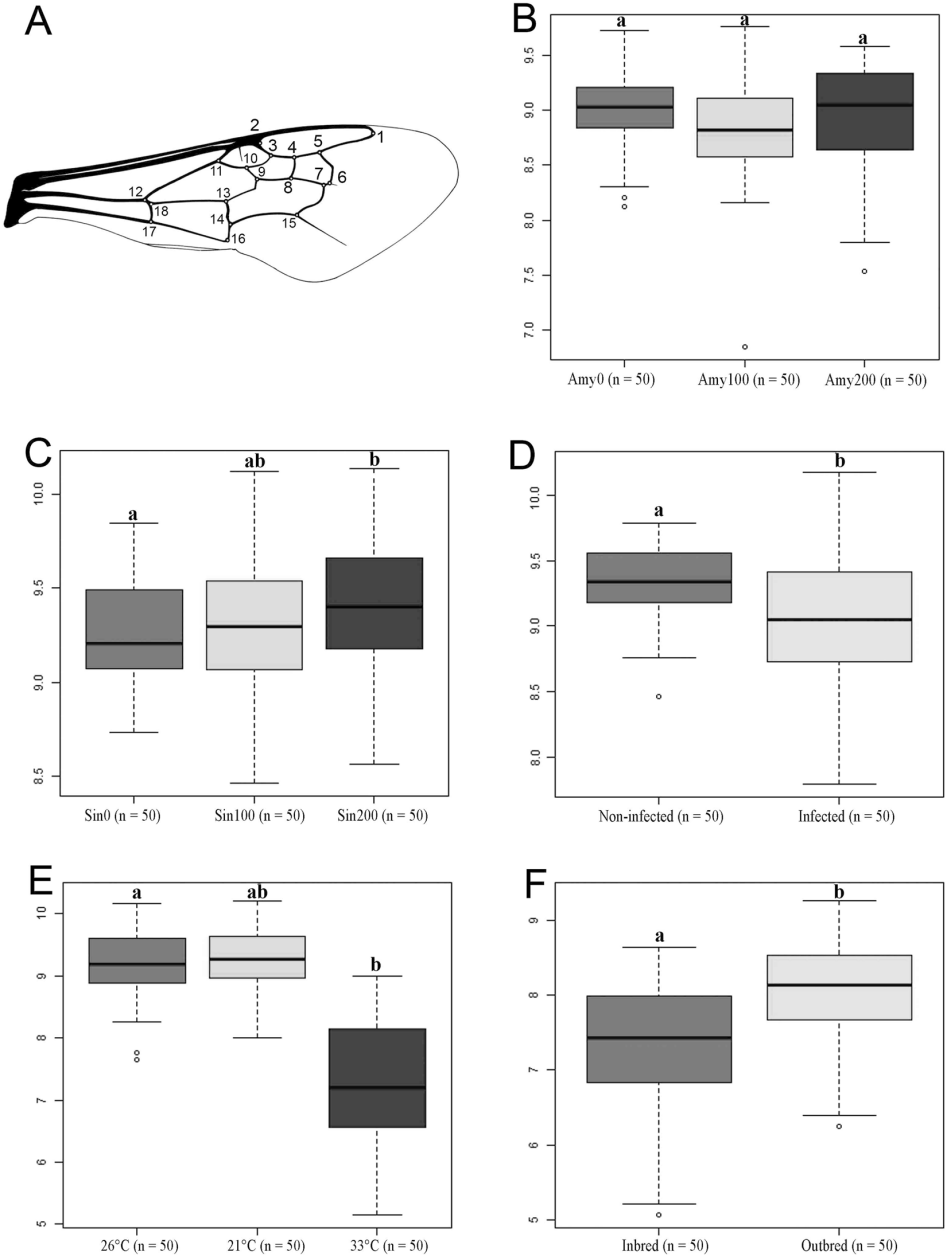
(**A**) Right forewing of *Bombus terrestris* with the 18 landmarks indicated to describe the shape. Length of radial cell is defined by the distance from landmark 1 to landmark 2. (**B–F**) Box plot of centroid size (CS) for the bumblebees bred in the five treatments: (**B**) Amygdalin treatment (Amy 0 = Amygdalin 0%, Amy100 = Amygdalin 100%, Amy200 = Amygdalin 200%). (**C**) Sinigrin treatment (Sin0 = Sinigrin 0%, Sin100 = Sinigrin 100%, Sin200 = Sinigrin 200%), (**D**) Parasite treatment, (**E**) Temperature treatment, (**F**). Inbreeding treatment. For each sub-dataset in each treatment, we measured 50 individuals.

Size - No significant difference in centroid size was found among diets supplemented in amygdalin and the control group (Fig. 1B; F_2,147_ = 1.36; p-value = 0.152). One-way ANOVA of the CS showed significant differences between the different sinigrin treatments (F_2,147_ = 3.75; p-value = 0.02). Bumblebees fed on a 200% sinigrin supplemented diet were significantly larger than those fed on the control diet (Fig. 1C; Tukey’s HSD test; F_1,49_ = 3.03; p-value = 0.02). No significant effect of 100% supplementation was found irrespective of the plant secondary compound (Tukey’s HSD test; all p-values > 0.05). Both the *A*. *bombi* infection (Fig. 1D; t-test; t = −4.49; df = 93.509; p-value < 0.001) and the inbreeding (Fig. 1F; t-test; t = 3.35; df = 78.487; p-value < 0.001) significantly reduced the bumblebee wing size. Regarding the thermic stress, one-way ANOVA showed significant differences between the different treatments (Fig. 1E; F_2,147_ = 127; p-value < 0.001). The males emerging from the micro-colonies reared at 33 °C were significantly smaller than those from the control micro-colonies (Tukey’s HSD test; F_1,49_ = 60.65; p-value < 0.001). By contrast, the cold exposure (i.e. thermic stress at 21 °C) had no significant impact (Tukey’s HSD test; F_1,49_ = 0.2; p-value > 0.05).

**Figure 2:**
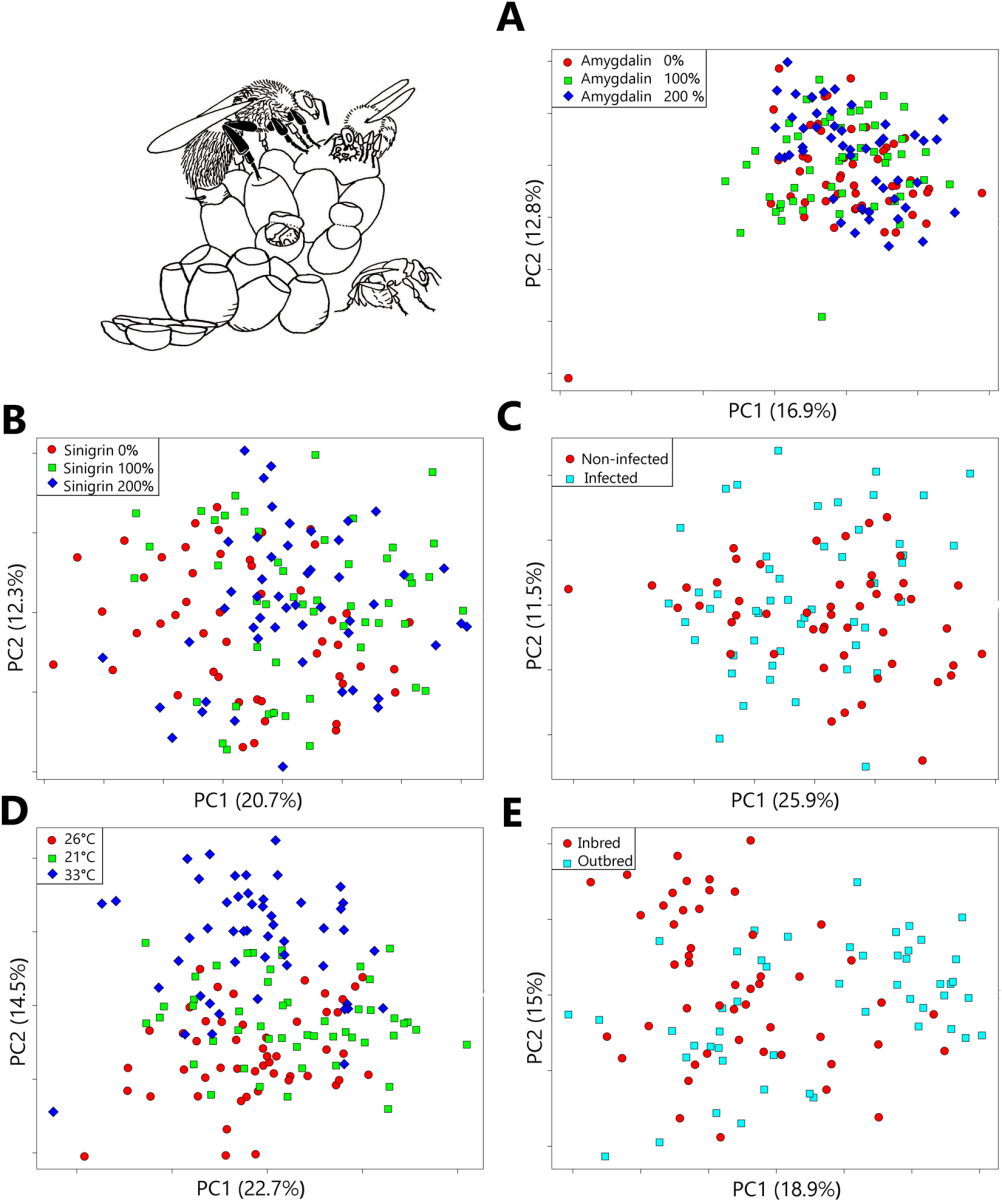
Ordination of the bumblebees bred in five treatments along the first two axes of the principal component analysis. (**A**) Amygdalin treatment; (**B**) Sinigrin treatment, (**C**) Parasite treatment, (**D**) Temperature treatment, (**E**) Inbreeding treatment. The percentage in brackets represents the variance along the first and the second axis of the PCA.

Shape - The PCA displayed large overlap of stressed and control groups, except for the inbreeding stress (i.e. slight overlap between inbred and outbred bumblebees; Fig. 2). The perMANOVA on the PCA axes revealed significant differences between each treatment inside each sub-dataset (Table 1). The between-group PCA (Fig. S1) including all experiments and treatments mostly opposes the different experiments (opposing for example the samples used in the inbreeding analysis to those from the sinigrin treatments on the PC1). No clustering was detected within the samples undergoing stressful treatments.

**Table 1:** Permanova on the PCA axes testing differences in wing shape of *Bombus terrestris* among each stressor. df: degree of freedom; MS: mean square estimates.

Stressor	Pairwise comparisons	df	MS	Statistics	R^2^	*p-value*
Amygdalin		2	0.00336	8.284	0.03	*0*.001**
	0 vs 100%	1	0.00154	3.673	0.02	*0*.001**
	0 vs 200%	1	0.00196	4.752	0.02	*0*.001**
	100 vs 200%	1	0.00191	4.696	0.02	*0*.001**
Sinigrin		2	0.00459	11.357	0.04	*0*.001**
	0 vs 100%	1	0.00304	7.277	0.04	*0*.001**
	0 vs 200%	1	0.0029	7.32	0.04	*0*.001**
	100 vs 200%	1	0.00104	2.603	0.01	*0*.01*
Thermic						
		2	0.00426	10.709	0.13	*0*.001**
	21 vs 26 °C	1	0.00167	41.717	0.04	*0*.001**
	21 vs 33 °C	1	0.00424	10.596	0.1	*0*.001**
	26 vs 33 °C	1	0.00687	17.485	0.15	*0*.001**
Parasitic		1	0.0029	7.291	0.02	*0*.001**
Infected vs control						
Inbreeding		1	0.00989	24.663	0.06	*0*.001**
Inbred vs outbred						

Variance of shape and size - Variance analyses revealed significant impact of several tested stresses (Table 2). Diet supplementation with amygdalin, either 100% or 200%, led to higher size variance than in control group (F_49,100_ = 2.5 and F_49,100_ = 8.59 respectively; p-value = 0.008 and p-value = 0.01 respectively) whereas diet supplementation with sinigrin did not show higher size variance compared to the control group (F-test; all p-value > 0.05). Wing size displayed a higher variance for individuals originating from infected micro-colonies (F_49,100_ = 3.54; p-value < 0.001) and inbred colonies (F_49,100_ = 1.82; p-value < 0.001) compared to their respective control group. Similarly, the thermic stress impacted the wing size variance with a higher variance for males reared at 33 °C (heat wave) than those reared at 21 °C and 26 °C (F_49,100_ = 1.35 and F_49,100_ = 1.74 respectively; df = 2; p-value < 0.001) but no significant differences in wing size variance were found between bumblebees reared at 21 °C and 26 °C (F_49,100_ = 1.02; p-value = 0.367). No significant differences in shape variance were found for any tested stress (F-test; all p-value > 0.05).

**Table 2:** Procrustes ANOVAS testing for differences in asymmetry in wing size and shape FA. MS: Mean Square df: degrees of freedom. *P < 0.05; **P < 0.01.

Trait	Amygdalin								
	0			100			200		
	df	MS	*F*	df	MS	*F*	df	MS	*F*
Size									
Individual	49	0.006619	1461.46**	49	0.013363	6571.13**	49	0.01281	2933.33**
Side	1	0.000026	5.88*	1	0.00001	4.27*	1	0.000025	5.82*
Side × Individual	49	0.000042	9.27**	49	0.000047	23.22**	49	0.00035	79.7**
Residuals	100	0.000005		100	0.000002		100	0.000004	
Shape									
Individual	1568	0.000039	1.85**	1568	0.000042	7.03**	1568	0.000041	6.49**
Side	32	0.00001	0.91*	32	0.000018	2.79**	32	0.000016	2.54**
Side × Individual	1568	0.000006	1.14**	1568	0.000005	5.75**	1568	0.000006	3.39**
Residuals	3200	0.000005		3200	0.000001		3200	0.000002	
	**Sinigrin**								
	**0**			**100**			**200**		
	**df**	**MS**	***F***	**df**	**MS**	***F***	**df**	**MS**	***F***
Size									
Individual	49	0.0042733	3430.28**	49	0.005119	3187.06**	49	0.005709	2562.66**
Side	1	0	0.02	1	0.000036	46.81**	1	0.000084	18.1**
Side × Individual	49	0.000045	36.06**	49	0.000048	15.76**	49	0.000028	24.24**
Residuals	100	0.000001		100	0.000002		100	0.000002	
Shape									
Individual	1568	0.000045	9.16**	1568	0.000046	8.54**	1568	0.00004	9.44**
Side	32	0.000017	3.4**	32	0.000021	3.97**	32	0.00002	5.25**
Side × Individual	1568	0.000005	5.16**	1568	0.000005	4.97**	1568	0.000004	4.26**
Residuals	3200	0.000001		3200	0.000001		3200	0.000001	
	**Temperature**								
	**26** **°C**			**21** **°C**			**33** **°C**		
	**df**	**MS**	***F***	**df**	**MS**	***F***	**df**	**MS**	***F***
Size									
Individual	49	0.012638	2951.08**	49	0.013951	2996.98**	49	0.073424	17510.9**
Side	1	0.000706	164.88**	1	0.000098	21.04**	1	0.000003	0.64
Side × Individual	49	0.000041	9.59**	49	0.000058	12.37**	49	0.00007	16.73**
Residuals	100	0.000004		100	0.000005		100	0.000004	
Shape									
Individual	1568	0.000137	7.58**	1568	0.000042	1.73**	1568	0.000039	5.81**
Side	32	0.000031	5.59**	32	0.000044	2.09**	32	0.00004	5.97**
Side × Individual	1568	0.000006	3.38**	1568	0.000006	1.1**	1568	0.000007	3.61**
Residuals	3200	0.000002		3200	0.000006		3200	0.000002	
	**Parasite**								
	**Control**			**Infected**					
	**df**	**MS**	***F***	**df**	**MS**	***F***			
Size									
Individual	49	0.003879	2163.44**	49	0.012683	10223.75**			
Side	1	0.000069	38.59**	1	0.000009	7.1**			
Side × Individual	49	0.000035	19.7**	49	0.000087	69.77**			
Residuals	100	0.000002		100	0.000001				
Shape									
Individual	1568	0.000048	8.52**	1568	0.00004	10.19**			
Side	32	0.000013	3.18**	32	0.000015	2.68**			
Side × Individual	1568	0.000005	5.91**	1568	0.000005	5.87**			
Residuals	3200	0.000001		3200	0.000001				
	**Inbreeding**								
	**Outbred**			**Inbred**					
	**df**	**MS**	***F***	**df**	**MS**	***F***			
Size									
Individual	49	0.036278	9897.85**	49	0.071714	31066.09**			
Side	1	0.000345	94.11**	1	0.000064	27.56**			
Side × Individual	49	0.000033	9.11**	49	0.000038	16.56**			
Residuals	100	0.000004		100	0.000002				
Shape									
Individual	1568	0.000037	2.16**	1568	0.000043	2.58**			
Side	32	0.000021	15.56**	32	0.000027	11.44**			
Side × Individual	1568	0.000007	0.56**	1568	0.000007	0.44**			
Residuals	3200	0.000012		3200	0.000016				

### Asymmetry analyses

In each group of each treatment, significant directional asymmetry (DA) was found in both wing size and shape (Table 1; Procrustes ANOVA, all p-value < 0.05), except for size DA of bumblebees bred in the control diet of the sinigrin treatment and those bred at a temperature of 33 °C (Procrustes ANOVA, both p-value > 0.05). When comparing groups, none tested stress impacted the shape DA (F-test, all p-value > 0.05) but significant differences in size DA were found for each stress (F-test, all p-value < 0.01). Regarding the nutritional stress, bumblebees fed on diet supplemented with sinigrin (both 100% and 200%) displayed a significantly higher size DA compared to the control group (F_1,100_ = 723.8 and F_1,100_ = 1872.4 respectively; df = 2; both p-value < 0.05) but no impact of amygdalin was detected (F-test; df = 2; p-value > 0.05). Whereas infested bumblebees displayed a higher size DA compared to non-infested ones (F_1,100_ = 5.43; df = 1; p-value < 0.001), those reared at 33 °C (i.e. thermic stress mimicking a heat wave) had a lower size DA compared to those exposed to cold and control groups (F_1,100_ = 33.05 and F_1,100_ = 252.96 respectively; df = 2; p-value < 0.001).

As for DA analysis, FA10 analysis did not show any difference in shape FA for the different tested stresses (Table 2; Procrustes ANOVA; p-value > 0.05). In contrast to shape FA, FA10 analysis revealed a significant increase of size FA in infested bumblebees (F_49,100 = _4.73; df = 1; p-value < 0.001) and in bumblebees reared at 33 °C (F_49,100_ = 5.93; df = 2; p-value = 0.03) compared to respective control groups. Regarding nutritional stress, size FA was significantly lower in bumblebees fed with a diet supplemented in 200% sinigrin compared to the other treatments (100% supplemented and control group), although the p-value was quite close to the threshold (F_49,100_ = 1.24 and F_49,100_ = 1.34 respectively; df = 2; p-value = 0.049 and 0.033 respectively). Size FA was not significantly different between the respective control groups and the amygdalin and inbreeding treatments (F-test; df = 2 and 1 respectively; all p-value > 0.05).

The PCoA applied on the shape FA matrices did not suggest any clustering of the matrices of the stressed samples (not shown).

## Discussion

There is evidence that secondary compounds e.g.^26^, parasites e.g.^27^, temperature e.g.^17^ and inbreeding e.g.^28^ may affect different morphological traits in their size and/or shape as confirmed in our results. While size and shape were always impacted by the tested stressors, size variance, size FA and DA were only impacted by some of them, and shape variance, FA and DA were never significantly impacted. Parasites and high rearing temperatures led to the strongest phenotype modifications: both impacted overall wing size and shape, size variance, size FA and DA, but also shape variance in the case of the parasitic stress. While in the literature, various stressors can induce unspecific and similar effects (e.g. on the immune system, metabolism or antioxidative response^29^), the effects can also be stressor-specific and differ for a same category of stressor (e.g. effects of secondary compounds on colony development^30^). In this paper, various stressful treatments did not generate similar shape changes (Fig. S1) which suggests that the nature of the treatment rather than stress *per se* determined the type of induced wing shape change. Similarly, for shape asymmetry, the lack of clustering of the FA matrices in the PCoA suggests that the nature of the shape asymmetry depends on the nature of the environmental treatment and that no general effect of stress can be detected.

Whereas food quantity and protein richness are known to have considerable effect on body size^31^, the impact of secondary compounds remains understudied. However there is evidence that several metabolic pathways can be affected by these secondary compounds^32^, which in turn can decrease the ability to buffer perturbations during the development although it has to be noticed that several secondary compounds can display beneficial effects on pollinators^33^. Our results suggested that such repercussions are probably compound-dependant as sinigrin and amygdalin displayed contrasted results, with a size increase for bumblebees fed on a diet supplemented in sinigrin and no significant effect for bumblebees fed on a diet supplemented in amygdalin. While several studies directly assessed the toxicity of amygdalin on bees e.g.^34^, the toxicity of sinigrin was only addressed in indirect studies on others insects^35^. Bees may have developed specific adaptation to those secondary compounds with the activation of detoxification mechanisms that could buffer perturbations of the developmental processes (i.e. only subtle modifications). Additionally the absence of significant effect of amygdalin on body size could result from an absence of biological targets in the process involved in wing development.

Although parasites can have a considerable impact on bees^36^ and interfere with their larval development^37^, the relationship among size, shape and parasitic prevalence has rarely been assessed^27^. Here we highlighted that infested bumblebees were significantly smaller than non-infested ones in the same rearing conditions. An interpretation of this effect is that the energy usually allocated towards the growth and development could be hijacked by the parasite and/or allocated to the immune response, thus leading to morphological changes and size decrease.

As predicted by the temperature-size (TS) rule, higher developmental temperature resulted in smaller body size. This TS rule is analogous to the Bergmann’s rule (i.e. larger body size at higher latitude) that applies to many ectotherms while mechanisms behind this trend remain debated^38^. However, recent studies provided opposite results, with bumblebees being smaller under higher latitudes and colder temperatures^39,40^. This converse Bergmann’s rule can result from a “season length effect” with starvation phenomenon (i.e. reduced availability of floral resources) that can occur at higher latitudes^41^. Such difference in larval feeding between *in natura* and laboratory conditions (i.e. fed *ad libitum*) could be responsible for those conflicting results. Moreover, since size does not increase monotonically with decreasing temperature, it would be more accurate to say that size is maximized at optimal physiological conditions.

Finally, while it is commonly assumed that inbreeding events affect several fitness components e.g.^42^, its impact on size is less consistently supported in the literature. In laboratory conditions, Gerloff and colleagues^43^ failed to detect any decrease in size after one generation of inbreeding using wild bumblebees. In our experiments, we used domesticated bumblebees obtained from industrial commercial breeding company that are likely to display lower levels of genetic diversity than wild ones^44^ since suffering from stronger inbreeding depression.

Regarding directional asymmetry and fluctuating asymmetry, no effect was detected on wing shape. By contrast, wing size asymmetry was significantly affected by the sinigrin, parasitic and thermic stresses; DA and FA being higher for the stressed individuals than for the control ones. With regard to FA, previous studies are inconsistent when assessing the impact of stress on developmental stability, whether studied organisms were insects or not^45,46^. For example, rearing temperature has repeatedly been found to affect FA in insects e.g.^47,48^, although some studies provided more equivocal or even negative results e.g.^49^. Extreme rearing temperature can affect the genetic cascade that is involved in the trait morphology during the larval development and could decrease the buffering developmental mechanisms of stabilizing proteins e.g.^50^. Additionally, inbreeding can lead to the expression of deleterious recessive mutations by increasing the homozygote frequency and thus could affect the developmental stability and increase FA^4^. Although the occurrence of diploid males in the inbred generation in our bioassays clearly demonstrated a genetic stress^51^, one generation of inbreeding might be insufficient to disrupt the bumblebee developmental stability, resulting in a similar FA between inbred and outbred colonies. Unfortunately, the lack of queens in the inbred generation prevented the production of additional inbred generations. Further bioassays are needed to test whether prolonged inbreeding may affect fluctuating asymmetry.

However, caution must be taken when considering the results based on FA as most of the groups displayed significant DA, which could lead to FA overestimation^52^. Genetic variation for DA could additionally inflate FA estimates e.g.^53^. Although DA has been widely reported in insects including bees e.g.^15,54^, it was not significant in most of the studies led on bee wings e.g.^55^. Actually, the use of FA as stress indicator seems to be traits-, stressors- and/or species-dependent, leading to non-congruent results among studies e.g.^46,56^. The setting of the study also influence this relationship. Indeed, different measures of FA can be used and display more or less accuracy to measure trait variability^56^. A review of environmental stressors^56^ even suggested that the nature of the stressor (e.g. temperature, nutrition, parasitism…) was not the main driver explaining the contrasting results among studies. By contrast, laboratory studies on fitness-related traits showed significantly larger negative effect of stressors on fluctuating asymmetry^56^. Even if our framework corresponds to those conditions, our results were still particularly contrasted.

Overall shape and size have been used in many fields such as Systematics and Palaeontology, mainly to detect changes among taxa or populations e.g.^57,58^. An additional application as stress estimator has been suggested since quantifying phenotype is less costly, can be non-lethal and is quite easy to obtain compared to other biomarkers (e.g. proteome^59^). Moreover, phenotypic changes occur usually before there is much mortality, which supports their use as a good stress marker for monitoring plan. Although fluctuating asymmetry has been used in various studies, the multiple responses in face of stressful conditions suggests that alternative phenotypic indicators of stress should be investigated. Our results have shown that size and/or shape are significantly affected by environmental and genetic stresses and are potentially accurate stress indicators^18^. Klingenberg and colleagues^60^ showed that variation within individuals was much lower than variation between individuals, corroborating the hypothesis that overall shape and size could be more sensitive stress indicators than FA. However, further studies are still needed to assess the accuracy of these stress indicators in additional taxa and their responses *in natura*.

## Methods

### Experimental design

*Bombus terrestris* (L., 1758) (see^61^ for an overview about this species) colonies were obtained from Biobest N.V. company (Westerlo, Belgium) and maintained in the laboratory of Zoology in Mons (Belgium).

Four different experimental bioassays were designed according to four tested stresses:

*Plant toxin stress* – We selected two secondary compounds detected in pollen collected by bumblebees: sinigrin and amygdalin. Sinigrin is a glucosinolate found in some Brassicaceae (2.226 µg/g in bee pollen samples^62^) and is toxic for most insects^63^. Amygdalin is a cyanogenic glycoside common in Fabaceae and Rosaceae (e.g. 1.889 mg/g in *Prunus amygdalus* pollen). When metabolized, it releases hydrogen cyanide (HCN) that is highly toxic for many animals and could impact bees through a long-term consumption^34^. This toxin induces “malaise” behaviour in honeybee, which consists in different behaviours like limited feeding and locomotion, and modifications of grooming behaviour^64^. Both molecules were detected in floral resources (i.e. pollen and nectar). We tested three diets per secondary compound: one control diet of willow pollen (*Salix* sp.)^65,66^ with water-ethanol mix; two diets of willow pollen (*Salix* sp.) added with the secondary compound (100% and 200% of their natural concentration in the pollen). We added some volume of BIOGLUC® syrup to each mixture to make consistent “candies” easier to manipulate. *Salix* pollen diet was prepared with commercial crushed honeybee pollen loads. For each treatment, ten queenless micro-colonies (i.e. colony containing only five workers^66^) were reared until emergence of at least five haploid males per micro-colony (n = 50 per treatment).

*Parasitic stress* – We used 20 queenless micro-colonies half of which have been infected with the neogregarine *Apicystis bombi* (Liu, Macfarlane & Pengelly, 1974) found in the body cavity of bumblebees^67^. This parasite appears to be highly virulent to bumblebee spring queens, inducing a rapid death through a pollen infested ingestion (i.e. *B*. *pratorum*^68^). Bumblebees were given a 20 μL droplet of syrup from a solution homogenised at 833 oocysts/μL. The 10 non-infected micro-colonies were used as a control. All micro-colonies were reared until emergence of at least five haploid males per micro-colony (n = 50 per treatment). Before the experiments the pollen diet was screened for the presence of *Apicystis bombi* following the protocol of^69^. No *Apicystis bombi* was detected in the pollen. *Thermic stress* – 30 queenless micro-colonies were reared at three different temperatures (21, 26 and 33 °C) in a climate controlled room. Bumblebee colonies are known to regulate their temperature to a set point between 27 and 33 °C^70^. Twenty one degrees corresponds to a cold stress: such low temperatures have indeed been shown to disrupt colony thermoregulation and have a negative impact on colony fecundity and brood incubation temperature^71^. Thirty three degrees is known as the upper limit where bumblebees can thermo-regulate their colonies by ventilation^70^ and is thus here considered a heat stress, mimicking the ground temperature during a heat wave. For each temperature, 10 micro-colonies were used. As for the previous experimental designs, all micro-colonies were reared until emergence of at least five haploid males per micro-colony (n = 50 per treatment).

*Inbreeding stress* – From the development of five colonies newly emerged queens and males were placed in a “flight cage” to force brother-sister mating. These mated queens were used to produce inbred generation colonies. During two months, the mated queens overwintered following the rearing method of ^72^. Fifteen out of 29 mated queens initiated colonies. For this inbreeding stress, we used workers (n = 50) to perform the subsequent morphometric analyses instead of haploid males because the inbred generation produced only few haploid males. Workers from the parent (outbred) generation were used as control (n = 50).

Our total dataset contains 650 individuals. The right and left forewings of each specimen were removed, placed on a slide and photographed using an Olympus SZH10 microscope with an AF-S NIKKOR 18–105 millimetres (Shinjuku, Japan) and GWH10X-CD oculars coupled with a Nikon D200 camera (Shinjuku, Japan).

### Wing size and shape analysis

Two-dimensional coordinates of 18 landmarks were obtained (Fig. 1A) from right and mirror-reflected left wings pictures using tps-DIG v2.30^73^. The landmark configurations were scaled, translated and rotated against the consensus configuration using the GLS Procrustes superimposition method, to remove all non-shape components^74^. The superimposition was performed using the package geomorph^75^. Wing size was estimated by the centroid size (CS, i.e. the square root of the sum of squared distance between all landmarks and their centroid; e.g.^76^). Each wing was digitized twice by the same experimenter (MG), in order to account for measurement error.

Preliminary analyses revealed that wing CS is related to wing size and was thus used as proxy. After having checked residuals normality (Shapiro test) and homoscedasticity (Bartlett test), type one ANOVAs were used to test the effect of the nutritional and thermic stresses on CS. Multiple pairwise comparisons (i.e. *post-hoc* tests, Tukey HSD) were conducted when significant difference among treatments was detected. T-tests were performed to assess the effect of parasitic and inbreeding stresses on CS.

For shape, principal component analyses (PCA) were conducted for each experiment to investigate the shape variation among the different treatments, using the geomorph function “plotTangentSpace”. Permutational MANOVAs and multiple pairwise comparisons were then performed on these principal components to assess the effect of each stressor on wing shape (Euclidean distances, 999 permutations, vegan function “adonis”) after having tested for multivariate homogeneity.

A global comparison of wing shape across experiments was done using a between-group PCA, taking each experiment as a priori grouping. This allowed us to investigate whether the various selected stressors had similar effects on wing shape.

### Asymmetry analyses

The procedure to measure FA (i.e. deviations from perfect symmetry by substracting left minus right sides values) is as follows. For shape, a two-way Procrustes ANOVA using individual, side, and their interaction as effects was applied to the superimposed coordinates. This Procrustes ANOVA allows, in a single procedure, to test for the significance of (i) the among-individual variation (individual effect), (ii) the directional asymmetry (DA) (side effect), and (iii) the fluctuating asymmetry (FA) relative to measurement error (interaction individual × side). The rationale for this procedure can be found in Palmer and Strobeck^5,77^ as well as in^78^. An error free estimate of shape FA was then derived (FA10 index^77^). Because FA10 is a variance estimate, F-test was used to compare differences in FA among the different treatments for each stressor. A similar two-way ANOVA was applied to centroid size to estimate size FA and an error corrected index (FA10) was computed. Inter-individual variances in size and shape were estimated as the mean squared (MS) of the individual effect, and the side MS was used as an estimate of directional asymmetry.

To investigate whether the patterns of shape asymmetry were similar across experiments and treatments (i.e. whether wings displayed the same ways to be asymmetric in response to different stressors), we performed a Principal Coordinates Analysis (PCoA) on the shape FA covariance matrices (i.e. interaction individual × side MSCP matrices; e.g.^49^).

Statistical analyses were performed using the software R version 3.4.0^79^ (2017, http://www.R-project.org/).

## Electronic supplementary material

Figure S1

## Data Availability

All morphometric data will be available on the Dryad database as TPS file once the paper will be accepted.
