# Supplementary material for: Stressful conditions reveal decrease in size, modification of shape but relatively stable asymmetry in bumblebee wings
Source: Sci Rep. 2018 Oct 11;8:15169. doi: 10.1038/s41598-018-33429-4 (PMC6181934; doi:10.1038/s41598-018-33429-4)

Stressful conditions reveal decrease in size, modification of shape but relatively stable asymmetry in bumblebee wings

Maxence Gerard[1*], Denis Michez[1], Vincent Debat[2], Lovina Fullgrabe[3], Ivan Meeus[4], Niels Piot[4], Ombeline Sculfort[2], Martin Vastrade[5], Guy Smagghe[4], Maryse Vanderplanck[1]

Figure captions

Figure S1. Ordination of the bumblebees bred in five treatments along the first two axes of the between-group principal component analysis. Amy0 = Amygdalin 0%, Amy100 = Amygdalin 100%, Amy200 = Amygdalin 200%; Sin0 = Sinigrin 0%, Sin100 = Sinigrin 100%, Sin200 = Sinigrin 200%; Inf = Infected bumblebees; Ctrl = Non-infected bumblebees; T1 = 26°C, T2 = 21°C, T3 = 33°C; G1 = outbred generation, G2 = inbred generation.

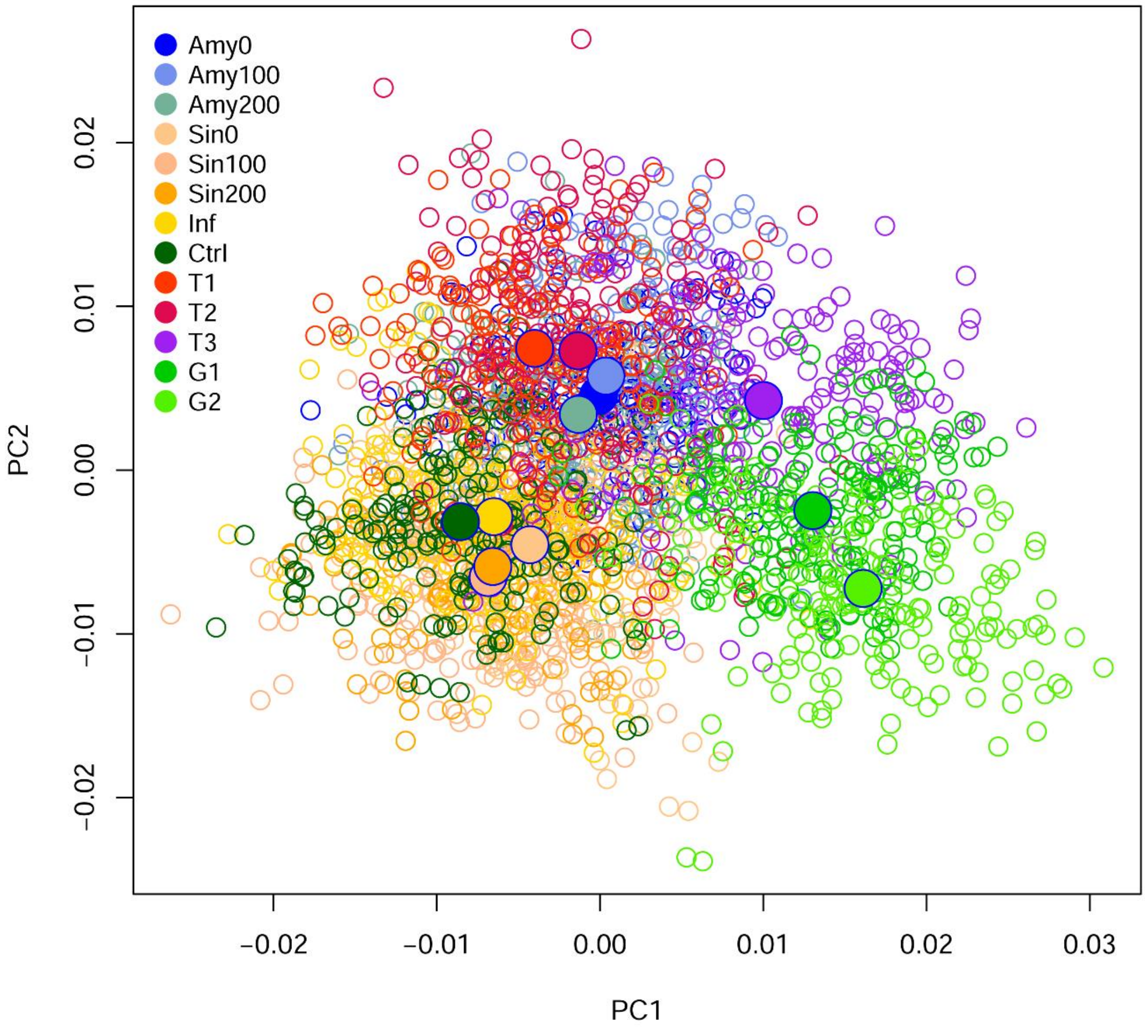

Supplement: Supplementary file 1 — Figure S1 [file 41598_2018_33429_MOESM1_ESM.pdf]
